# Cholestatic pruritus: a knowledge update^☆^

**DOI:** 10.1016/j.abd.2021.06.007

**Published:** 2022-03-09

**Authors:** Thaís Reginatto Nietsche, Gabriel Dotta, Carlos Baptista Barcaui, Maria Lúcia Cardoso Gomes Ferraz

**Affiliations:** a Hospital São Paulo, Universidade Federal de São Paulo, São Paulo, SP, Brazil; b Hospital Universitário Pedro Ernesto, Universidade do Estado do Rio de Janeiro, Rio de Janeiro, RJ, Brazil

**Keywords:** Cholestasis, Dermatology, Pruritus

## Abstract

This review is focused on updating knowledge about cholestatic pruritus. It summarizes clinical-epidemiological characteristics, pathophysiology, diagnostic approach, and evidence-based therapeutic recommendations regarding this form of pruritus. Pruritus is a frequent symptom that accompanies several liver diseases, particularly cholestatic ones. The symptom may be mild and tolerable, but it can also dramatically reduce the quality of life. Although the exact pathophysiology of this form of pruritus remains unclear, current evidence supports a mixed origin. It is extremely important for dermatologists to have knowledge about cholestatic pruritus since they are usually the first physicians to be sought by the patient when they experience the symptom. In the absence of specific dermatological alterations, cholestasis must always be considered as a possible cause of pruritus. In addition to allowing an adequate diagnosis, a better pathophysiological understanding of hepatic pruritus provides the identification of new therapeutic targets and, consequently, optimization of the approach in patients with this condition.

## Introduction

Cholestasis is defined as a decrease in biliary flow secondary to impaired secretion by hepatocytes or the obstruction of this flow in the intra- or extra-hepatic bile ducts. On the other hand, the clinical description of cholestasis is any condition in which substances normally excreted in the bile are retained. Serum levels of conjugated bilirubin and bile salts are the most commonly measured ones.^1^

The cutoff levels of serum alkaline phosphatase (AP) and gamma-glutamyl transferase (GGT) that characterize cholestasis and require diagnostic investigation are debated: AP values ​​greater than 1.5 times the upper limit of normal (ULN) and GGT levels three times the ULN.^2^

Clinically, the impaired bile flow can present as fatigue, osteoporosis, fat malabsorption, clotting disorders, jaundice, and pruritus.^3^

Pruritus is a frequent symptom that accompanies several liver diseases, particularly cholestatic ones. It can be mild and tolerable, but it can also dramatically reduce the quality of life, cause considerable sleep deprivation, depressive symptoms, and induce suicidal ideation in the most severely affected patients.^4^

Because it is a subjective sensation whose intensity is difficult to estimate, cholestatic pruritus still receives little attention from some patients and, unfortunately, from many physicians. This happens because, very often, it is mistakenly considered a condition that is not worth, in fact, assessing or treating. Nevertheless, evidence suggests that it is a symptom with a substantial impact on the quality of life of patients with cholestatic liver disease.^5^

## Associated diseases and epidemiology

**Figure 1:**
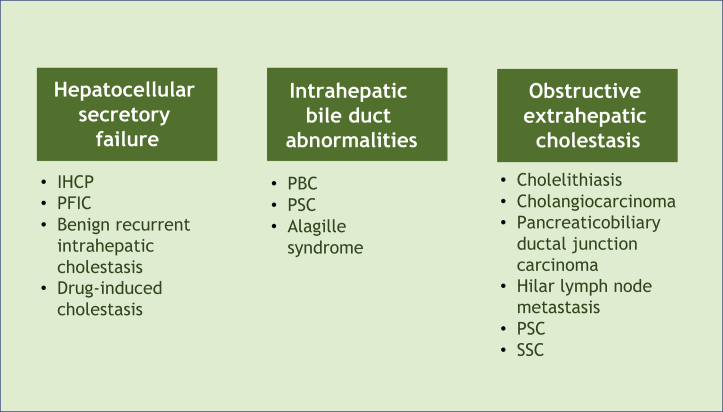
Classification of cholestasis according to its origin in the biliary system and corresponding examples. PBC, Primary Biliary Cholangitis; PSC, Primary Sclerosing Cholangitis; SSC, Secondary Sclerosing Cholangitis, IHCP, Intrahepatic Cholestasis of Pregnancy; PFIC, Progressive Familial Intrahepatic Cholestasis.

Pruritus can develop in patients with cholestasis related to any etiology. Cholestasis is classified according to its origin at different levels of the biliary system. The main affected sites and their respective examples are hepatocellular secretory failure (intrahepatic cholestasis of pregnancy, progressive familial intrahepatic cholestasis, benign recurrent intrahepatic cholestasis, drug-induced cholestasis); intrahepatic bile duct abnormalities (primary biliary cholangitis, primary sclerosing cholangitis, Alagille syndrome), and extra-hepatic obstructive cholestasis (cholelithiasis, primary and secondary sclerosing cholangitis, cholangiocarcinoma, pancreaticobiliary ductal junction carcinoma, hilar lymph node metastasis) (Fig. 1).^6^ All these conditions, therefore, represent potential diseases associated with cholestatic pruritus.

It should be noted that the prevalence of pruritus in different cholestatic liver diseases varies considerably. It is, for instance, the main symptom of intrahepatic cholestasis of pregnancy (IHCP). It occurs in 70% to 80% of patients with primary biliary cholangitis (PBC) and primary sclerosing cholangitis (PSC), and its frequency decreases with disease progression. This symptom is also present in 16% to 45% of obstructive cholestasis from calculus or tumor (Table 1).^7^ Overall, pruritus secondary to liver disease is more frequently seen in intrahepatic than extra-hepatic cholestatic diseases.^8^

**Table 1:** Prevalence of pruritus in the main cholestatic diseases.

Disease	Prevalence of pruritus
IHCP	100%
PBC/PSC	70%–80%
Lithiasis/Tumor	16%–45%

PBC, Primary Biliary Cholangitis; PSC, Primary Sclerosing Cholangitis; IHCP, Intrahepatic Cholestasis of Pregnancy.

## Pathophysiology

The pathophysiological mechanism of cholestatic pruritus is still not well defined. Several hypotheses have been raised in the past. The most important one included the participation of bile acids and endogenous opioids.^9^

### Bile acids

Bile acids have been implicated in the pathogenesis of cholestasis pruritus. They accumulate in the tissues of patients with liver disease and – under experimental conditions that included intracutaneous injections in normal volunteers – bile acids have been reported to produce local “itching”. The injection of substances into the skin, however, is not a model for studying the pruritus caused by cholestasis. The idea that bile acids cause pruritus in cholestasis was further perpetuated by a report of four patients with PBC who experienced pruritus after ingestion of synthetic bile acid. Three observations, however, do not tend to support the fact that these substances cause pruritus in cholestasis. First, as patients with liver disease progress to failure, bile acid levels become extremely high, and the pruritus tends to cease. Second, despite showing marked elevations in serum bile acid levels, not all patients with cholestasis report pruritus. Third, this symptom fluctuates regardless of serum bile acid levels.^10^

### Endogenous opioids

The pruritic effects of opioids, on the other hand, vary depending on the activity of their receptor subtypes. Endogenous opioids can activate μ-opioid receptors (MOR) to induce pruritus, while incitement of κ-opioid receptors (KOR) inhibits it. An imbalance between MOR (increased) and KOR (decreased) has been suggested in conditions associated with pruritus, such as end-stage renal disease and cholestasis. As the liver disease progresses, liver clearance of endogenous opioids decreases, with a consequent increase in their serum levels. However, pruritus severity does not correlate with endogenous opioid levels.^11^

### Lysophosphatidic acid (LPA) and autotaxin (ATX)

Findings indicate that LPA, a potent neuronal activator, as well as ATX, the enzyme that produces LPA, are key elements in the pathophysiology of pruritus in cholestasis. Serum ATX activity correlates with pruritus intensity and response to treatment in patients with cholestatic pruritus, but not with this symptom when it is associated with other etiologies.^4^, ^12^, ^13^, ^14^, ^15^, ^16^, ^17^ Carrion et al. (2018) found in patients with cholestasis and pruritus that ATX activity is significantly higher than in those with cholestasis without pruritus, and ATX levels correlate with the severity of this symptom. Additionally, the response to therapeutic interventions (bile acid-binding resins or rifampicin) is associated with decreased serum autotaxin activity, further supporting the role of autotaxin-LPA in the pathogenesis of pruritus in cholestasis.^11^

**Figure 2:**
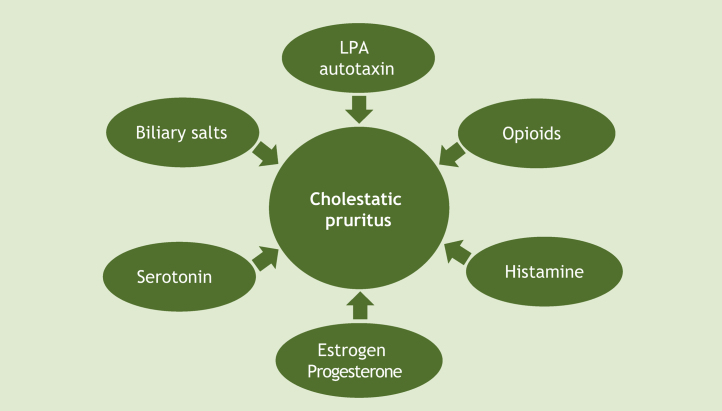
Pathophysiology of cholestatic pruritus. LPA, Lysophosphatidic Acid.

Given all these hypotheses, it is worth noting that there has been great progress in recent decades regarding the understanding of cholestatic pruritus mechanisms. Although the exact pathophysiology of this type of pruritus remains unclear, current evidence supports a mixed origin (Fig. 2).^14^ At the tissue level, as already mentioned, local deposition of excess bile salts was the first suggested mechanism for pruritus in cholestatic disease. To date, the proposed modulators of the pruritic mechanism of cholestasis include bile salts, opioids, histamine and lysophosphatidic acid-LPA (through autotaxin-ATX), progesterone, estrogens, and serotonin. The best current evidence suggests a complex interaction of these substances, and so there are therapeutic agents for multiple targets.^18^

## Clinical manifestations

Cholestatic pruritus can occur at any stage of liver disease. Its intensity is often exacerbated by psychological stress, heat, and contact with wool. Cold temperatures often produce symptom relief. Moreover, pruritus is more common in women.^19^

Pruritus associated with cholestasis often exhibits a circadian rhythm, with patients reporting greater intensity in the early hours of the night. This type of pruritus is usually reported to be most pronounced on the palms of the hands and soles of the feet, but it can be generalized and range from mild to severe. In contrast to the pruritus seen in dermatological disorders, primary skin lesions are not detectable in these patients. However, intense scratching can cause secondary alterations, such as excoriations, folliculitis, lichenification, and prurigo nodularis, which occasionally lead to a misinterpretation of its etiology as a primary skin condition.^20^, ^21^

Determining the intensity of pruritus as objectively as possible is extremely relevant, not only for research purposes but also in clinical practice, for adequate therapeutic follow-up. However, there is no ‘one-size-fits-all’ scoring system. Currently, there are several assessment methods for estimating the severity of pruritus: the measurement of the sensory threshold or the act of excoriating and the one-dimensional pruritus severity scales, for instance, the Visual Analog Scale (VAS), the Numerical Scale (NS), and the Verbal Assessment Scale, in addition to multidimensional questionnaires such as the 5-D Itch Scale (5D-IS), the Pruritus Severity Scale, and the Eppendorf Pruritus Questionnaire.^22^

Based on recently published consensuses, the use of VAS in daily clinical practice and of at least two independent methods in research studies or clinical trials is recommended. However, no widely accepted, standardized, and validated questionnaire for objective measurement of pruritus is available.^23^, ^24^

A more accurate method of objectively assessing pruritus is to use the excoriating activity monitoring system, which involves applying piezoelectric film technology to generate a scratch transducer that is attached to a fingernail, allowing the recording of the act of scratching. Irrespective of limb movement during long periods, for instance, 24 hours. These instruments were designed and validated.^25^

## Diagnosis

A presumptive diagnosis of cholestatic pruritus can be made in patients with cholestasis who complain of itching. However, when there is no known cause of cholestasis, it should be considered that approximately one in five patients with generalized pruritus has a systemic disease.^26^

In this context, there is a long list of differential diagnoses for the causes of pruritus, such as skin diseases, systemic diseases (lymphoma and other neoplasms, uremia and chronic renal failure, hypothyroidism, diabetes, iron deficiency anemia), psychogenic, neurological, and iatrogenic pruritus, for instance, caused by drugs. At least a detailed skin examination should be performed to look for primary skin changes related to dermatological disorders, in addition to adequate patient history, physical examination, and a comprehensive laboratory evaluation, which may include imaging tests, to rule out, among other conditions, myeloproliferative diseases or uremic pruritus in end-stage renal disease, considering that hepatobiliary disorders, chronic kidney disease, and hematological disorders are the three most common systemic conditions associated with chronic pruritus.^8^, ^19^

The initial evaluation should include complete blood count (CBC), thyroid hormone, urea, and creatinine levels, chest X-ray, and markers of cholestasis and liver disease, including alkaline phosphatase, gamma-GT, as well as bilirubin and aminotransferase levels.^27^

It is important to consider that clinical signs suggestive of chronic liver diseases, such as jaundice, palmar erythema, and telangiectasias, are only seen in a minority of patients, as pruritus often occurs in the early stages of liver disease. Therefore, attention should be paid to the possibility of cholestatic pruritus when a patient presents with chronic pruritus without specific dermatological signs.^20^

## Treatment

**Figure 3:**
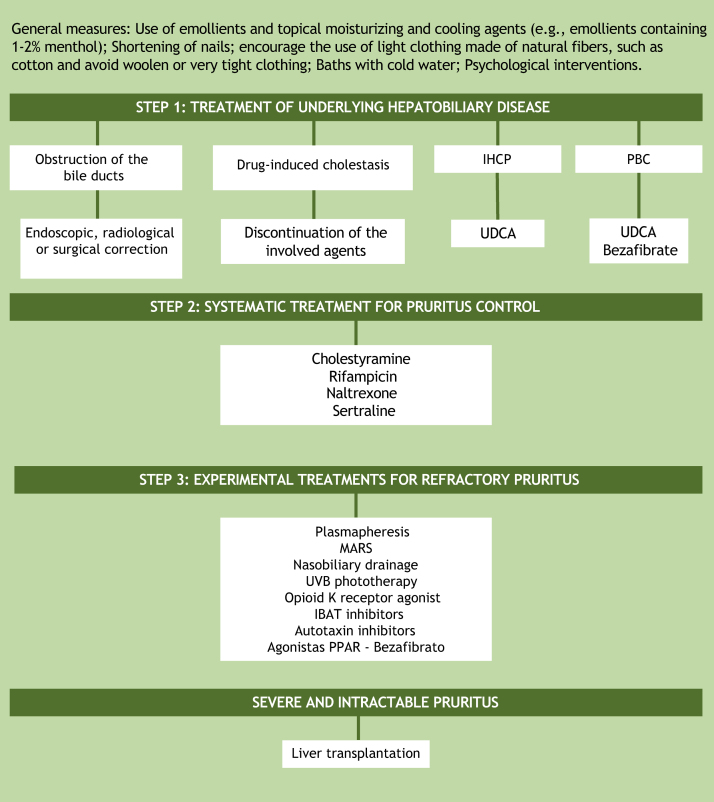
Algorithm for the treatment of cholestatic pruritus. UDCA, Ursodeoxycholic Acid; PBC, Primary Biliary Cholangitis; IHCP, Intrahepatic Cholestasis of Pregnancy; IBAT, Ileal Bile Acid Transporter; MARS, Molecular Adsorbent Recirculating System, PPARs, Peroxisome Proliferator-Activated Receptor Agonists; UVB, Ultraviolet radiation type B.

Assuming that the unknown pathogenesis of cholestatic pruritus is one of the factors preventing the development of effective therapy, it is understood why treatment options are limited and still do not provide relief for all patients. A practical recommendation for the step-by-step management of this symptom is based on the guidelines of the European Association for the Study of the Liver (EASL) and the American Association for the Study of Liver Diseases (Fig. 3).^28^, ^29^ The proposal, as the first step in the treatment of patients with cholestatic pruritus, is to treat the underlying hepatobiliary disease and ruling out bile duct obstruction. In cases of bile duct obstruction, endoscopic, radiological, or surgical correction should be performed. In drug-induced cholestasis, discontinuation of the implicated agents is the treatment of choice. In intrahepatic cholestasis of pregnancy and primary biliary cholangitis, ursodeoxycholic acid (UDCA) is the first treatment option. UDCA is a disease-modifying therapy, but there is no evidence that it has any effect on pruritus, except in intrahepatic cholestasis of pregnancy. In PBC patients who do not respond to UDCA alone, bezafibrate can be used.^2^, ^19^ Currently, peroxisome proliferator-activated receptor agonists (PPARs) such as bezafibrate have also been considered as an experimental treatment option for refractory cholestatic pruritus.^8^

If the underlying hepatobiliary disease cannot be corrected, systematic treatment should be initiated, aiming at controlling pruritus in a gradual approach, as suggested in the aforementioned guideline recommendations for the management of pruritus in cholestatic patients.^28^, ^29^ There are four objectives in cholestatic pruritus therapy. The first one is to remove substances associated with pruritus from the enterohepatic cycle through bile acid sequestrants (cholestyramine). The second objective is to control the metabolism of some factors associated with pruritus by the pregnane X receptor (PXR), such as rifampicin, an enzyme inducer. The third and fourth objectives are related to the perception of pruritus. This is the case of µ-opioid antagonists and selective serotonin reuptake inhibitors (SSRIs), respectively.^2^ The suggested doses of these medications are shown in Table 2. It is important to note that all the aforementioned drugs, except for cholestyramine, are considered “off-label” in the management of cholestatic pruritus. Although frequently prescribed in clinical practice, antihistamines are generally ineffective in hepatic pruritus.^30^, ^31^

**Table 2:** Therapeutic recommendations for cholestatic pruritus.

Drug	Dose
Cholestyramine	4–16 g/day
Rifampicin	150–600 mg/day
Naltrexone	12,5–50 mg/day
Sertraline	75–100 mg/day

Adapted from Düll et al.^30^

In the management of treatment-refractory pruritus, removal of possible factors associated with the symptom from the systemic circulation using the molecular adsorbent recirculating system (MARS) or plasmapheresis may be attempted.^14^ Newer agents under consideration for the treatment of cholestatic pruritus include ileal bile acid transporter (IBAT) and autotaxin inhibitors. IBAT represents an interesting therapeutic target for the interruption of the enterohepatic cycle, thus increasing the secretion of bile salts by the intestinal tract. Examples of IBAT inhibitors are linerixibat, maralixibat, and odevixibat. Another medication for pruritus control is the κ-opioid receptor agonist, nalfurafine, which has been approved in Japan for the treatment of cholestatic pruritus since 2015. Physical approaches such as nasobiliary drainage and ultraviolet B (UVB) radiation phototherapy constitute experimental treatments, with case reports showing benefits, but without randomized controlled trials yet.^28^, ^29^, ^32^

In addition to systemic treatment, general skincare guidelines should be given to patients. These include using emollients and topical moisturizing and cooling agents (e.g. emollients containing 1%–2% menthol), shortening of fingernails, encouraging the use of light clothing made of natural fibers such as cotton, avoiding woolen and clothing that is too tight. Cold water baths in situations where the pruritus is aggravated by heat and psychological interventions can also be useful.^28^, ^32^

## Conclusion

Pruritus is a common symptom in many dermatological conditions, and therefore, dermatologists are usually the first physicians to evaluate patients with this symptom. In this context, it is extremely important for these professionals to have knowledge about the systemic conditions related to it, particularly the one addressed in the present article, cholestasis. Therefore, chronic pruritus in the absence of specific skin alterations should always encourage the request for laboratory markers of cholestasis.

It should be noted that the greater the understanding on this subject, the greater the chances of developing effective therapies for cholestatic pruritus and, consequently, improving the quality of life of these patients.

## Financial support

None declared.

## Authors’ contributions

Thais Reginatto Nietsche: Design and planning of the study; data collection, or analysis and interpretation of data; drafting the article or critical review of important intellectual content; collection, analysis and interpretation of data; critical review of the literature.

Gabriel Dotta: Drafting of the manuscript or critical review of important intellectual content; collection, analysis, and interpretation of data.

Carlos Baptista Barcaui: Design and planning of the study; approval of the final version of the manuscript.

Maria Lúcia Cardoso Gomes Ferraz: Design and planning of the study; effective participation in research orientation; approval of the final version of the manuscript.

## Conflicts of interest

None declared.

## References

[1] Nazer H. (2017). Medscape [Internet].

[2] European Association for the Study of the Liver (2009). EASL Clinical Practice Guidelines: management of cholestatic liver diseases. J Hepatol.

[3] Chazouille`res O., Housset C., Rode’s J., Benhamou J.P., Blei A., Reichen J., Rizzetto M., Dufour J.F. (2007). Textbook of Hepatology: From Basic Science to Clinical Practice.

[4] Beuers U., Kremer A.E., Bolier R., Elferink R.P.J.O. (2014). Pruritus in cholestasis: facts and fiction. Hepatology.

[5] Jin X.Y., Khan T.M. (2016). Quality of life among patients suffering from cholestatic liver disease-induced pruritus: A systematic review. J Formos Med Assoc.

[6] Bolier R., Elferink R.P.J.O., Beuers U. (2013). Advances in pathogenesis and treatment of pruritus. Clin Liver Dis.

[7] Kremer A.E., Elferink R.P.J.O., Beuers U. (2011). Pathophysiology and current management of pruritus in liver diseases. Clinics Res Hepatol Gastroeneterol.

[8] Düll M.M., Kremer A.E. (2019). Treatment of Pruritus Secondary to Liver Disease. Curr Gastroenterol Rep.

[9] Elferink R.P.J.O., Kremer A.E., Beuers U. (2011). Mediators of pruritus during cholestasis. Curr Opin Gastroenterol.

[10] Bergasa N.V. (2018). The pruritus of cholestasis: From bile acids to opiate agonists: Relevant after all these years. Med Hypotheses.

[11] Carrion A.F., Rosen J.D., Levy C. (2018). Understanding and Treating Pruritus in Primary Biliary Cholangitis. Clin Liver Dis.

[12] Beuers U., Kremer A.E., Bolier R., Elferink R.P.J.O. (2014). Pruritus in cholestasis: facts and fiction. Hepatology.

[13] Reich A., Szepietowski J.C. (2016). Measurement of Itch Intensity. Curr Probl Dermatol.

[14] Kremer A.E., Bolier R., Dijk R., Elferink R.P.J.O., Beuers U. (2014). Advances in pathogenesis and management of pruritus in cholestasis. Dig Dis.

[15] Nakanaga K., Hama K., Aoki J. (2010). Autotaxin – an LPA producing enzyme with diverse functions. J Biochem.

[16] Kremer A.E., Dijk R., Leckie P., Schaap F.G., Kuiper E.M.M., Mettang T. (2012). Serum autotaxin is increased in pruritus of cholestasis, but not of other origin, and responds to therapeutic interventions. Hepatology.

[17] Finlay A.Y., Khan G.K. (1994). Dermatology Life Quality Index (DLQI) – a simple practical measure for routine clinical use. Clin Exp Dermatol.

[18] Khanna A., Leighton J., Wong L.L., Jones D.E. (2018). Symptoms of PBC – Pathophysiology and management. Best Pract Res Clin Gastroenterol.

[19] Vloo C.D., Nevens F. (2019). Cholestatic pruritus: an update. Acta Gastroenterol Belg.

[20] (2016). Mittal A. Cholestatic Itch Management. Curr Probl Dermatol..

[21] Hegade V.S., Kendrick S.F.W., Rehman J., Jones D.E. (2015). Itch and liver: management in primary care. Br J Gen Pract.

[22] Reich A., Bożek A., Janiszewska K., Szepietowski J.C. (2017). 12-Item Pruritus Severity Scale: Development and Validation of New Itch Severity Questionnaire. Biomed Res Int.

[23] Ständer S., Augustin M., Reich A., Blome C., Ebata T., Phan N.Q. (2013). Pruritus assessment in clinical trials: consensus recommendations from the international fórum for the study of itch (IFSI) special interest group scoring itch in clinical trials. Acta Derm Venereol.

[24] Weisshaar E., Gieler U., Kupfer J., Furue M., Saeki H., Yosipovitch G. (2012). Questionnaires to assess chronic itch: a consensus paper of the special interest group of the International Forum on the Study of Itch. Acta Derm Venereol.

[25] Jones E.A., Bergasa N.V. (2000). Evolving concepts of the pathogenesis and treatment of the pruritus of cholestasis. Can J Gastroenterol.

[26] Kremer A.E., Feramisco J., Reeh P.W., Beuers U., Elferink R.P.J.O. (2014). Receptors, cells and circuits involved in pruritus of systemic disorders. Biochim Biophys Acta.

[27] Yosipovitch G., Bernhard J.D. (2013). Clinical practice. Chronic Pruritus. N Engl J Med.

[28] European Association for the Study of the Liver (2017). EASL Clinical Practice Guidelines: The diagnosis and management of patients with primary biliary cholangitis. J Hepatol.

[29] Lindor K.D., Bowlus C.L., Boyer J., Levy C., Mayo M. (2019). Primary Biliary Cholangitis: 2018 Practice Guidance from the American Association for the Study of Liver Diseases. Hepatology.

[30] Dull M.M., Kremer A.E. (2018). Management of Chronic Hepatic Itch. Dermatol Clin.

[31] Hönig S., Herder B., Kautz A., Trautwein C., Kremer A.E. (2018). Pruritus strongly reduces quality of life in PBC patients – real life data from a large national survey. J Hepatol.

[32] Dull M.M., Kremer A.E. (2020). Newer Approaches to the Management of Pruritus in Cholestatic Liver Disease. Current Hepatology Reports.

